# Simvastatin Preserves Human Microvascular Endothelial Barrier Function Through Mevalonate-Dependent Regulation of RhoB Localization and Activation

**DOI:** 10.64898/2026.10.28.762609

**Published:** 2026-10-30

**Authors:** Alejandra Morales-Maldonado, Jordan S. Pober, Richard W. Pierce

## Abstract

Endothelial barrier failure contributes to edema, tissue hypoperfusion, and organ dysfunction during inflammatory critical illness. RhoB has emerged as a regulator of tight junction disruption in human microvascular endothelial cells, but whether clinically used drugs can modulate RhoB-dependent barrier failure remains unclear. We tested whether simvastatin preserves human dermal microvascular endothelial cell barrier function during TNF stimulation and examined the role of mevalonate-dependent RhoB localization and activation. Simvastatin pretreatment attenuated TNF-induced loss of transendothelial electrical resistance and preserved junctional ZO-1 and claudin-5 organization. Simvastatin reduced membrane-associated and increased nuclear RhoB, decreased active RhoB, and suppressed myosin light chain phosphorylation. RhoB silencing phenocopied the barrier-protective effect of simvastatin, and combined RhoB silencing and simvastatin did not provide additive protection. Mevalonate supplementation reversed simvastatin-mediated preservation of barrier function, restored peripheral RhoB localization, and restored MLC phosphorylation. These findings identify mevalonate-dependent RhoB spatial regulation as a statin-sensitive mechanism contributing to inflammatory microvascular barrier dysfunction.

## Full Text Availability

The license terms selected by the author(s) for this preprint version do not permit archiving in PMC. The full text is available from the preprint server.

